# Protein disulfide isomerase PDI8 is indispensable for parasite growth and associated with secretory protein processing in *Toxoplasma gondii*

**DOI:** 10.1128/mbio.02051-24

**Published:** 2024-08-20

**Authors:** Chaoyue Wang, Pei Sun, Yonggen Jia, Xinming Tang, Xianyong Liu, Xun Suo, Hongjuan Peng

**Affiliations:** 1 Department of Pathogen Biology, Guangdong Provincial Key Laboratory of Tropical Diseases Research, School of Public Health, Southern Medical University, Guangzhou City, Guangdong Province, China; 2 Key Laboratory of Infectious Diseases Research in South China (Ministry of Education), Southern Medical University, Guangzhou, Guangdong, China; 3 Guangdong Key Laboratory of Animal Conservation and Resource Utilization, Institute of Zoology, Guangdong Academy of Science, Guangzhou, Guangdong Province, China; 4 Beijing Institute of Tropical Medicine, Beijing Friendship Hospital, Capital Medical University, Beijing, China; 5 Institute of Animal Science, Chinese Academy of Agricultural Sciences, Beijing, China; 6 National Key Laboratory of Veterinary Public Health Security, Key Laboratory of Animal Epidemiology and Zoonosis of Ministry of Agriculture, National Animal Protozoa Laboratory & College of Veterinary Medicine, China Agricultural University, Beijing, China; University of Geneva, Geneva, Switzerland

**Keywords:** *Toxoplasma gondii*, protein disulfide isomerase, invasion, secretory protein, protein processing

## Abstract

Protein disulfide isomerase, containing thioredoxin (Trx) domains, serves as a vital enzyme responsible for oxidative protein folding (the formation, reduction, and isomerization of disulfide bonds in newly synthesized proteins) in the endoplasmic reticulum (ER). However, the role of ER-localized PDI proteins in parasite growth and their interaction with secretory proteins remain poorly understood. In this study, we identified two ER-localized PDI proteins, TgPDI8 and TgPDI6, in *Toxoplasma gondii*. Conditional knockdown of TgPDI8 resulted in a significant reduction in intracellular proliferation and invasion abilities, leading to a complete block in plaque formation on human foreskin fibroblast monolayers, whereas parasites lacking TgPDI6 did not exhibit any apparent fitness defects. The complementation of TgPDI8 with mutant variants highlighted the critical role of the CXXC active site cysteines within its Trx domains for its enzymatic activity. By utilizing TurboID-based proximity labeling, we uncovered a close association between PDI proteins and canonical secretory proteins. Furthermore, parasites lacking TgPDI8 showed a significant reduction in the expression of secretory proteins, especially those from micronemes and dense granules. In summary, our study elucidates the roles of TgPDI8 and sets the stage for future drug discovery studies.

**IMPORTANCE:**

Apicomplexans, a phylum of intracellular parasites, encompass various zoonotic pathogens, including *Plasmodium*, *Cryptosporidium*, *Toxoplasma*, and *Babesia*, causing a significant economic burden on human populations. These parasites exhibit hypersensitivity to disruptions in endoplasmic reticulum (ER) redox homeostasis, necessitating the presence of ER-localized thioredoxin (Trx) superfamily proteins, particularly protein disulfide isomerase (PDI), for proper oxidative folding. However, the functional characteristics of ER-localized PDI proteins in *Toxoplasma gondii* remain largely unexplored. In this study, we identified two ER-localized proteins, namely, TgPDI8 and TgPDI6, and demonstrated the indispensable role of TgPDI8 in parasite survival. Through a comprehensive multi-omics analysis, we elucidated the crucial role of TgPDI8 in the processing of secretory proteins in *T. gondii*. Additionally, we introduced a novel ER-anchored TurboID method to label and identify canonical secretory proteins in *T. gondii*. This research opens up new avenues for understanding oxidative folding and the secretory pathway in apicomplexan parasites, laying the groundwork for future advancements in antiparasitic drug development.

## INTRODUCTION

In eukaryotic cells, the folding of nascent peptides into native proteins involves the formation of disulfide bonds, a process known as oxidative protein folding. The endoplasmic reticulum (ER) serves as the primary site for oxidative protein folding, predominantly mediated by protein disulfide isomerase (PDI) (1). Disulfide bond formation primarily occurs in secretory proteins localized within the lumen of secretory organelles. The accumulation of misfolded proteins and perturbation of ER redox homeostasis can trigger ER stress, influencing protein processing (2, 3). Therefore, organisms rely on the collaborative efforts of various PDI proteins to guarantee efficient and accurate disulfide bond formation. The number of PDIs varies among higher eukaryotes, for instance, the human and Arabidopsis genomes contain over 20 PDIs and more than 14 PDIs, respectively (4–6). Most members of the PDI family feature at least one thioredoxin (Trx) domain with a conserved “CXXC” active site that is essential for the reduction, oxidation, or isomerization of disulfide bonds in misfolded proteins.

*Toxoplasma gondii*, an intracellular protozoan parasite capable of invading almost all nucleated cells, presents a serious threat to pregnant women and immunocompromised patients (7). The parasite secretes unique proteins rich in disulfide bonds from organelles such as rhoptries, micronemes, and dense granules (8). The functional roles of ER-localized PDIs have been elucidated in *Plasmodium* (9). Using activity-based crosslinkers, PfJ2 substrates including PfPDI8 have been identified as crucial for asexual growth (10). Previous studies in *T. gondii* have revealed localization of members of the Trx superfamily to various cellular compartments, including the apicoplast, cytosol, and ER. Two apicoplast thioredoxins (ATrx1 and ATrx2) participate in distinct apicoplast biogenesis pathways, with TgATrx1 implicated in ER-to-apicoplast trafficking and TgATrx2 in regulating apicoplast gene expression (11, 12). Cytoplasmic proteins containing Trx domains have also been characterized in *T. gondii*, such as CTrp26, the deletion of which showed no discernible impact on parasite growth (13). Investigations on ER-localized PDIs in *T. gondii* remain limited; however, studies have demonstrated that immunization with recombinant TgPDI8 effectively reduces parasite load, highlighting their importance as immunogenic antigens (14). Treatment of *Toxoplasma* tachyzoites with the PDI inhibitor KSC-34 results in reduced parasite attachment to host cells (15), similar to findings in *Neospora caninum*, a closely related coccidian parasite (16). However, comprehensive functional characterization of these ER-resident proteins in *T. gondii* remains largely unexplored.

Here, we provide a comprehensive characterization and functional validation of two ER-localized PDI proteins, TgPDI8 and TgPDI6. Conditional knockdown of TgPDI8 resulted in a serve growth defect. Additionally, we observed a robust association between ER-localized PDI proteins and secretory proteins (MICs, ROPs, and GRAs), with altered expression levels observed in some secretory proteins upon TgPDI8 depletion. TurboID-based proximity labeling significantly enriched these secretory proteins using TgPDI6 as bait (17). Overall, our findings highlight the critical role of TgPDI8 in parasite growth and the processing of secretory proteins.

## RESULTS

### TgPDI8 and TgPDI6 are both localized to the endoplasmic reticulum

**Fig 1:**
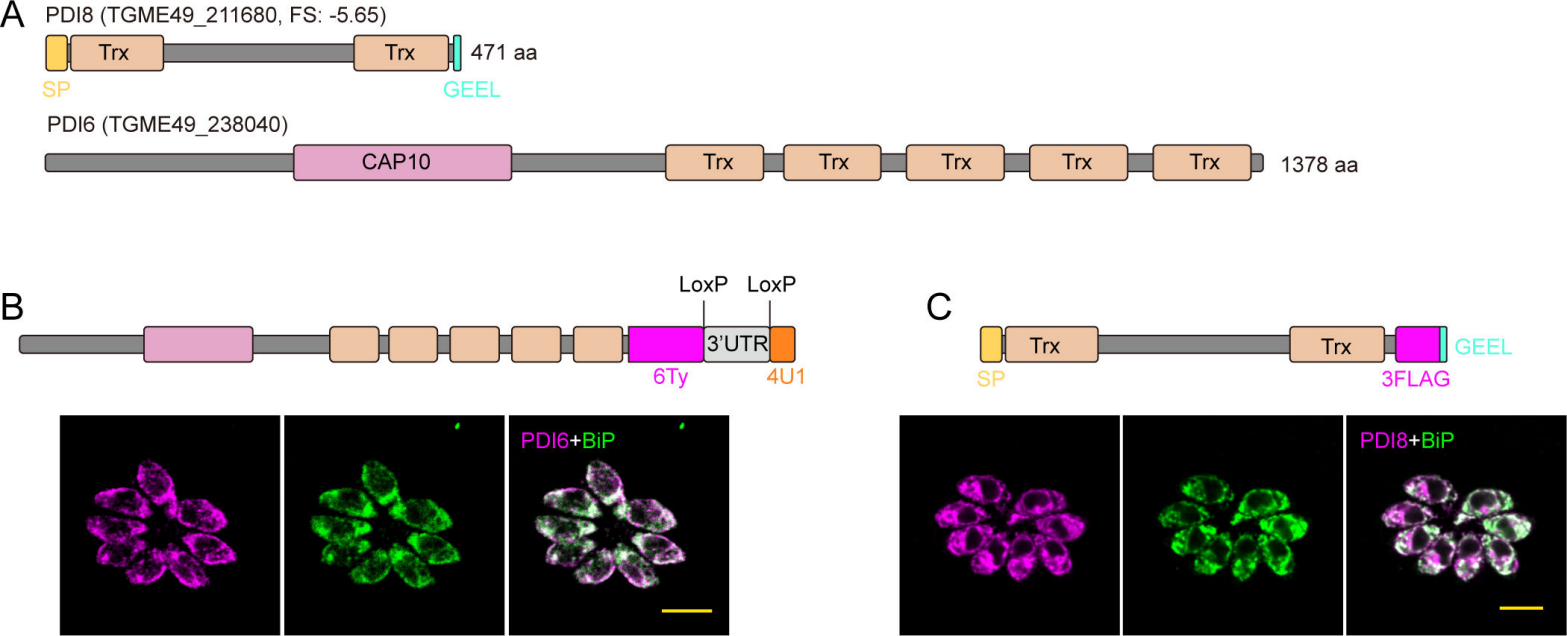
Subcellular localization of TgPDI6 and TgPDI8 in *T. gondii.* (**A**) Schematic representation of the structure characteristics of two protein disulfide isomerases (TgPDI6 and TgPDI8). FS, fitness score of genes from a recent whole-genome CRISPR analysis (19). (**B and C**) Immunofluorescence assays (IFAs) of PDI6-6Ty-4U1 (**B**) and PDI8-3FLAG (**C**) parasites using anti-Ty antibodies (magenta, **B**) or anti-FLAG antibodies (magenta, **C**). Anti-BiP antibodies served as an ER marker (green, **B and C**). Scale bars = 5 µm.

Based on hyperLOPIT data (18), we identified two PDIs potentially localized to the ER: TgPDI8 (TGME49_211680) and TgPDI6 (TGME49_238040). Both TgPDI8 and TgPDI6 belong to the Trx superfamily, characterized by multiple Trx domains (Fig. 1A). The presence of conserved cysteine residues (CXXC) within each Trx domain indicates their potential involvement in disulfide oxidoreductase/isomerase activities. TgPDI8 exhibits a low phenotypic score (−5.65) in the *Toxoplasma* genome-wide CRIPSR screen (19), suggesting the crucial importance of this protein for parasite fitness. Phylogenetic and protein domain architecture analysis confirmed the conservation of TgPDI8 across apicomplexan parasites (Fig. S1A through C).

To explore the localization of TgPDI8 and TgPDI6, we generated epitope-tagged lines in DiCre RH parasites with a C-terminal 6Ty-4U1 tag. TgPDI6 exhibited perfect ER localization, whereas TgPDI8 showed perinuclear labeling that partially overlapped with BiP (Fig. 1B; Fig. S1D). Furthermore, a strong fluorescent signal was detected in the parasitophorous vacuole (PV) of TgPDI8 (Fig. S1D). Notably, a C-terminal GEEL motif at positions 417 to 420 in the TgPDI8 protein sequence resembles the canonical ER retention signal H/KDEL, commonly involved in retrieving soluble proteins to the ER. We hypothesized that the insertion of a C-terminal 6Ty tag might disrupt the localization of TgPDI8. To investigate this possibility, we introduced the ER retention signal (GEEL) following the 6Ty tag, yet TgPDI8 was still secreted into the PV (Fig. S1E). However, by utilizing a 3×FLAG tag followed by the GEEL sequence to label the TgPDI8, we confirmed its correct ER localization through IFA (Fig. 1C). Overall, both TgPDI8 and TgPDI6 are localized to the ER.

### TgPDI8 is indispensable for tachyzoite growth *in vitro*

**Fig 2:**
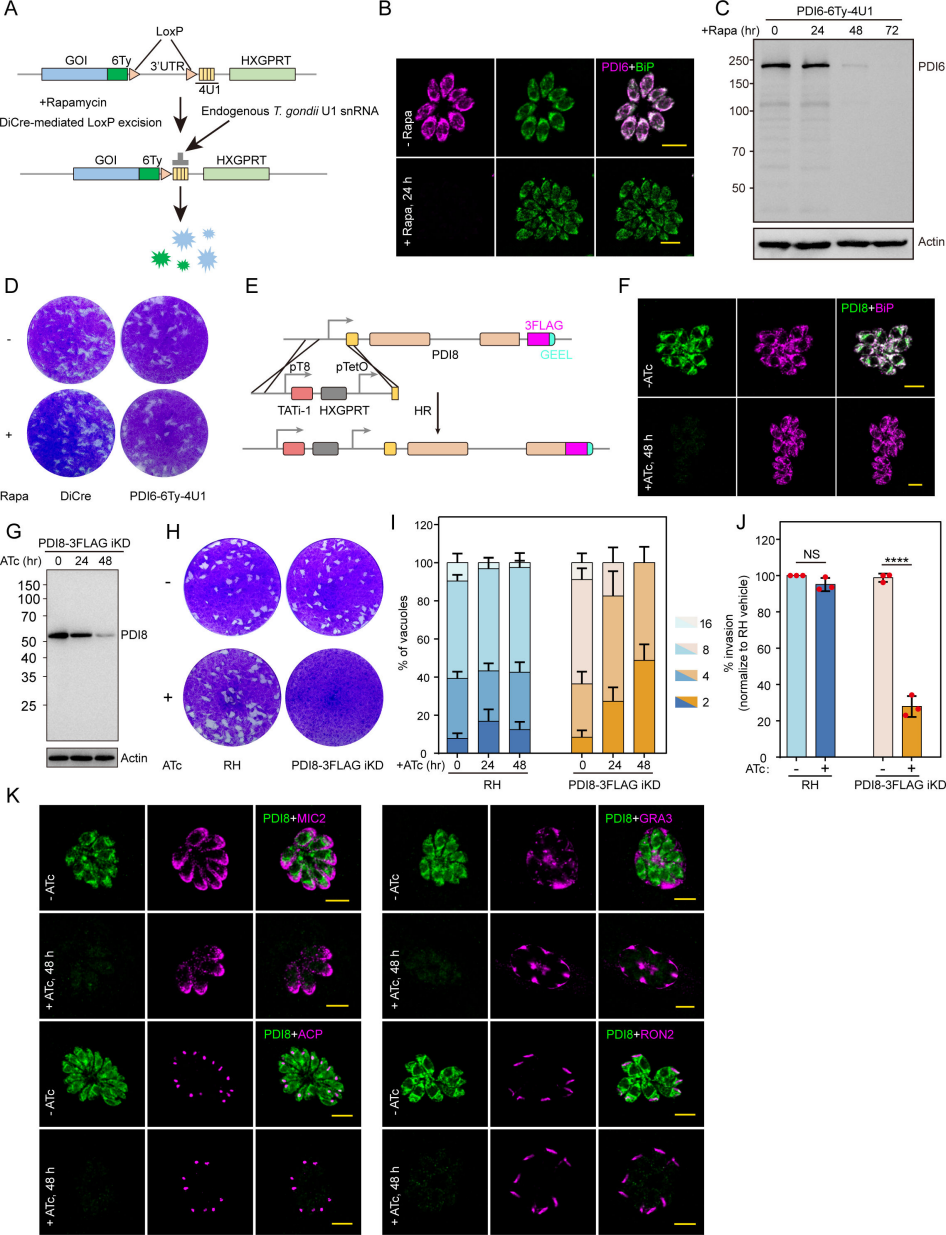
TgPDI8 is necessary for the lytic life cycle of *T. gondii in vitro.* (**A**) Schematic representation of TgPDI6 gene expression constructs engineered for the UI knockdown strategy in the DiCre strain. (**B**) IFAs of intracellular PDI6-6Ty-4U1 parasites after 24-h treatment with rapamycin or dimethyl sulfoxide (DMSO). Scale bars = 5 µm. (**C**) Western blot analysis of TgPDI6 expression levels after 24, 48, and 72 h of rapamycin treatment. Antibodies against TgActin were used as a loading control. (**D**) Plaque formation by DiCre and PDI6-6Ty-4U1 parasites on human foreskin fibroblast (HFF) monolayers treated with rapamycin or DMSO for 7 days. (**E**) Schematic representation of CRISPR/Cas9-mediated integration of the Tet-inducible knockdown system into the 5′UTR of the PDI8-3FLAG parasites. (**F**) IFAs of intracellular PDI8-3FLAG iKD parasites after 48 h in the presence of anhydrotetracycline (ATc) or the vehicle (DMSO). Scale bars = 5 µm. (**G**) Western blot analysis of the expression of TgPDI8 after 24 and 48 h of ATc treatment. Antibodies against TgActin were used as a loading control. (**H**) Plaque formation by RH, PDI8-3FLAG iKD parasites growing on HFF monolayers treated with ATc or DMSO for 7 days. (**I**) Intracellular replication of RH and PDI8-3FLAG iKD parasites after treatment with ATc or DMSO for 0, 24, and 48 h. Similar results were obtained from three independent assays. (**J**) Invasion of RH and PDI8-3FLAG iKD parasites on HFF monolayers following 48 h of treatment with ATc or DMSO. Statistical significance was assessed using an unpaired *t*-test (ATc vs DMSO), ****P < 0.0001. (**K**) IFAs of PDI8-3FLAG iKD parasites after 48 h without (−) or with (+) ATc treatment, using multiple organelle antibodies: anti-MIC2 (micronemes, magenta), anti-ACP (apicoplast, magenta), anti-GRA3 (dense granules, magenta), and anti-RON2 (rhoptries, magenta). Scale bars = 5 µm.

To investigate the function of TgPDI6, we utilized the U1 snRNP-mediated gene silencing system in DiCre-expressing parasites (20, 21). Upon rapamycin induction, Cre-mediated recombination was activated, resulting in the excision of the 3′-UTR and positioning of the four U1 recognition sites directly after the stop codon, leading to pre-mRNA degradation (Fig. 2A). After treating parasites with rapamycin, we successfully confirmed the depletion of TgPDI6 through IFA (Fig. 2B) and immunoblotting (Fig. 2C). The knockout of TgPDI6 had no impact on parasite fitness, as demonstrated by plaque formation (Fig. 2D). Furthermore, the complete knockout of TgPDI6 further confirmed its non-essential role in parasite growth (Fig. S2A through C).

To assess the function of TgPDI8, we first generated an epitope-tagged TgPDI8 line with an auxin-inducible degron and three HA tags (AID-3HA) at the C-terminus in TIR1 RH parasites (22). IFA using anti-BiP antibodies as an ER marker revealed colocalization of TgPDI8 with BiP (Fig. S2D). However, attempts to deplete TgPDI8 using auxin proved unsuccessful, indicating a non-functional AID system for TgPDI8 depletion (Fig. S2E). We therefore generated a conditional knockdown strain using the Tet-inducible system in the PDI8-3FLAG background (Fig. 2E). We observed co-localization of TgPDI8 with BiP and detected reduced TgPDI8 expression upon ATc induction, confirmed by IFA and immunoblotting (Fig. 2F and G). Knockdown of TgPDI8 resulted in significant growth defects, particularly evident in plaque formation (Fig. 2H), replication capacity (Fig. 2I), and invasion capability (Fig. 2J). To assess the impact on various organelles, we examined the morphologies of the ER, micronemes, apicoplast, dense granules, and rhoptries following TgPDI8 depletion. Notably, the organelle morphologies remained unchanged in TgPDI8-depleted parasites (Fig. 2F and K). In summary, these findings underscore the indispensable role of TgPDI8 in the lytic cycle of *T. gondii*.

### Functional analysis of the CXXC active site cysteines of TgPDI8

**Fig 3:**
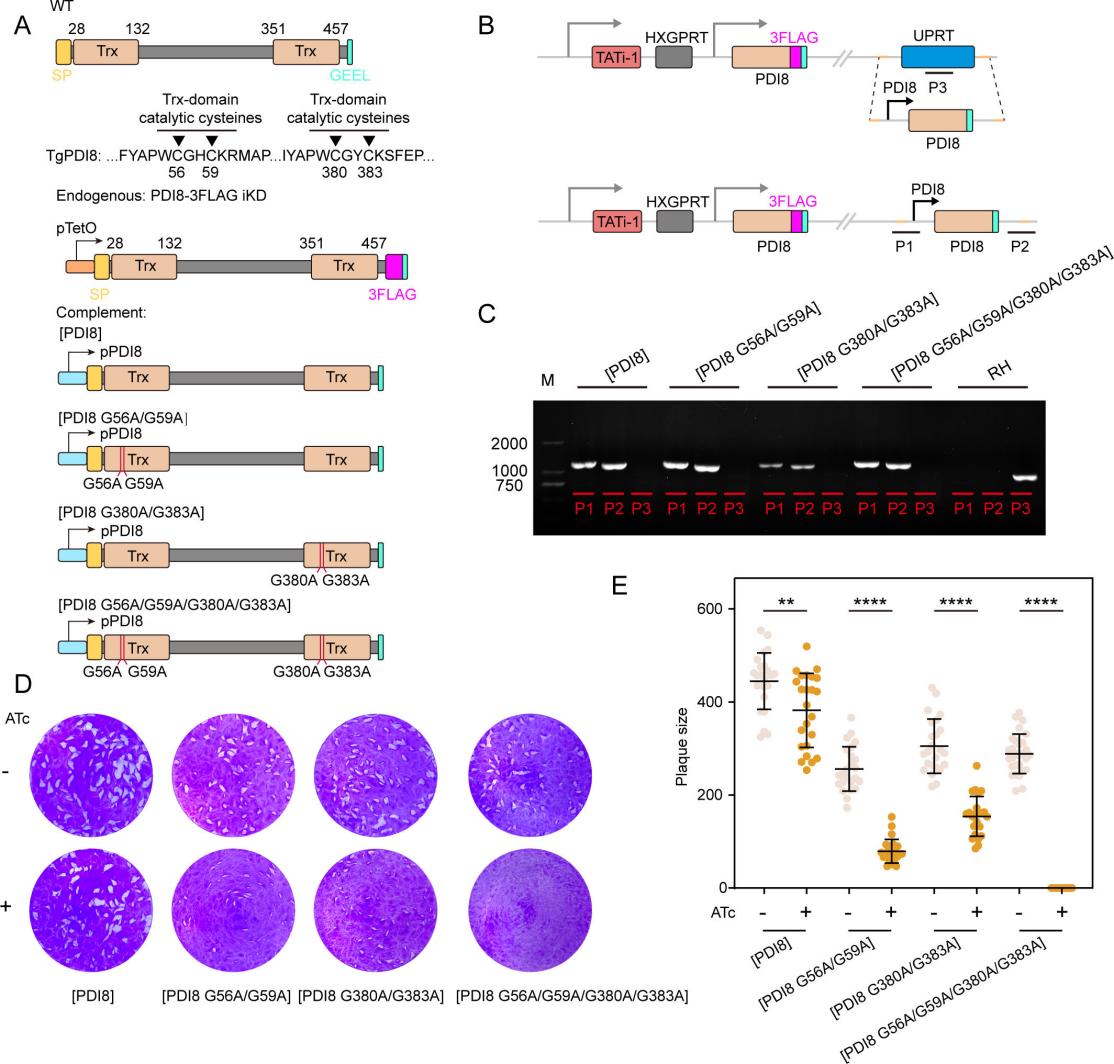
Essential function of two classical CXXC active site cysteines in Trx domains for TgPDI8 activity. (**A**) Schematic representation of introducing a second copy of wild-type or mutant isoforms of TgPDI8, free of any tags, into the UPRT locus in the regulatable PDI8-3FLAG iKD background, driven by the native TgPDI8 promoter. (**B**) Strategy for generating wild-type or mutant TgPDI8-complemented parasites. (**C**) Diagnostic PCR confirming integration and gene complementation of wild-type or mutant TgPDI8. (**D**) Plaque formation by wild-type or mutant TgPDI8-complemented lines growing on HFF monolayers for 7 days in the absence or presence of ATc. (**E**) Quantification of plaques corresponding to D, mean ± SD (*n* = 3). Statistical evaluation was conducted using an unpaired *t*-test (ATc versus DMSO), **P < 0.01 and ****P < 0.0001.

TgPDI8 consists of two Trx domains with classical CXXC active site cysteines, facilitating its function as an oxidoreductase/isomerase in oxidative folding. To determine the functional importance of these CXXC active site cysteines, we first expressed a second copy of wild-type TgPDI8 fused with 6HA upstream of the GEEL sequence, driven by the native TgPDI8 promoter, into the regulatable PDI8-3FLAG iKD background (Fig. S3A). The expression of the second copy of wild-type TgPDI8 yielded identical band sizes, as confirmed by western blot analysis (Fig. S3B). However, the complemented line ([PDI8-6Ty]) exhibited perinuclear labeling that partially colocalized with BiP, accompanied by a strong fluorescent signal in the PV (Fig. S3C). Phenotypic analysis revealed that [PDI8-6Ty] parasites formed smaller plaques on host cell monolayers after ATc-induced depletion of endogenous TgPDI8 (Fig. S3D and E). The incomplete restoration of parasite growth by ectopic expression of wild-type TgPDI8 may be attributed to mislocalization when fused with a 6Ty tag. To explore this further, we introduced tag-free ectopic expression of wild-type TgPDI8 in the regulatable PDI8-3FLAG iKD background, confirmed by diagnostic PCR and sequencing (Fig. 3A through C). Phenotypic analysis demonstrated that the tag-free line effectively rescued the growth defect, thereby confirming the adverse effect of 6Ty-induced mislocalization on PDI8 functionality (Fig. 3D and E; Fig. S3D and E). Based on this, we subsequently generated three tag-free PDI8 mutant complemented lines, including variants with mutations in the first CGHC catalytic site ([PDI8 G56A/G59A]), the second CGYC catalytic site ([PDI8 G380A/G383A]), and both sites ([PDI8 G56A/G59A/G380A/G383A]) (Fig. 3A and B). The successful generation of these three TgPDI8 mutant-complemented lines was confirmed via diagnostic PCR and sequencing (Fig. 3C). Mutations in either CXXC catalytic site led to a significant growth defect characterized by smaller plaque formation, whereas mutation in both sites completely blocked plaque formation (Fig. 3D and E). In conclusion, these findings demonstrate the crucial functional significance of CXXC active site cysteines for TgPDI8 enzymatic activity.

### ER-localized PDIs are closely associated with secretory proteins

**Fig 4:**
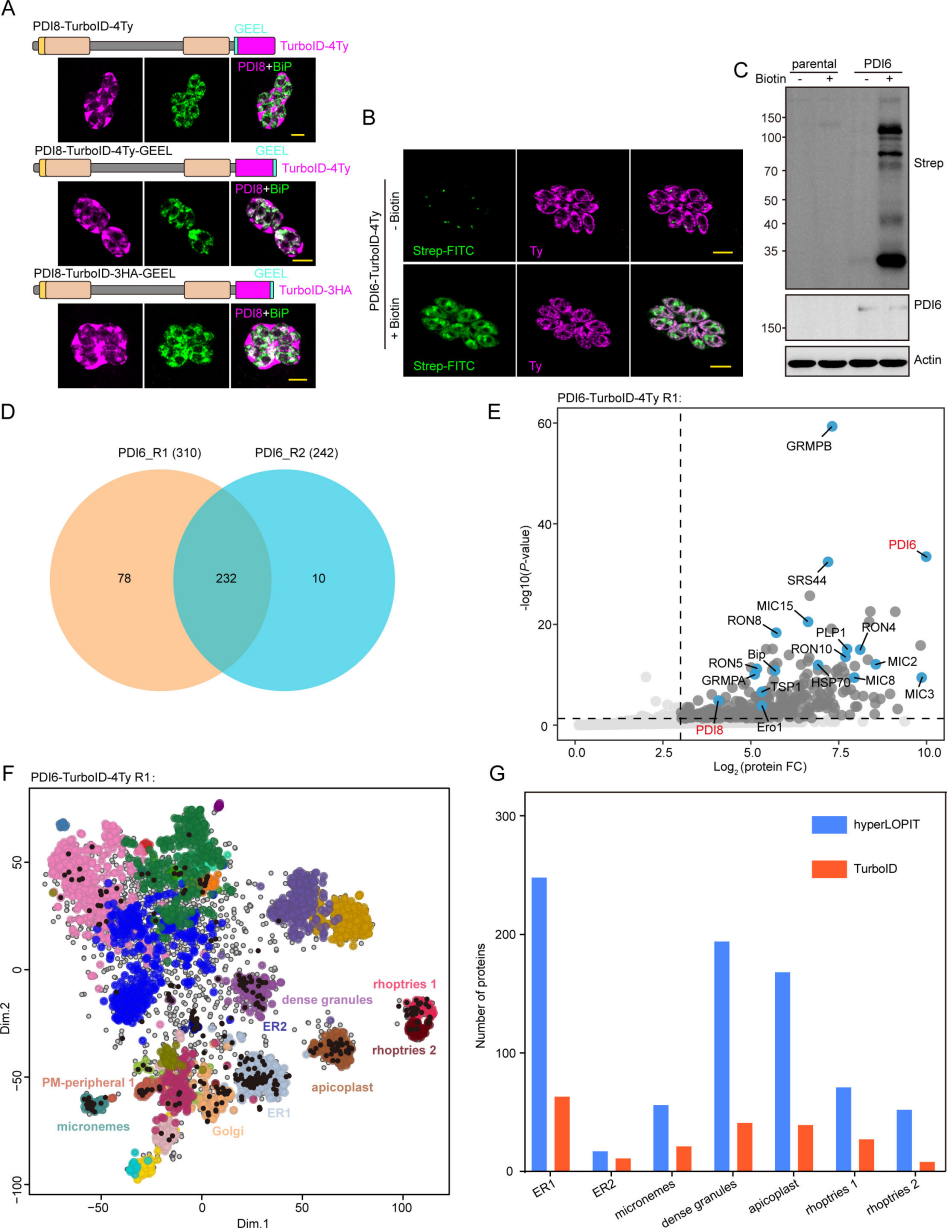
Identification of proximal and interacting proteins using endoplasmic reticulum-localized TgPDI6 protein as bait. (**A**) IFAs of PDI8-TurboID-3HA-GEEL and PDI8-TurboID-4Ty-GEEL parasites using anti-HA (or anti-Ty) antibodies (magenta) and anti-BiP antibodies (green). Scale bars = 5 µm. (**B**) IFA detection of biotinylated proteins in PDI6-TurboID-4Ty parasites. The subcellular localization of TgPDI6 was detected using anti-Ty antibodies (magenta), and biotinylated proteins were detected using streptavidin-FITC (green). Scale bar = 5 µm. (**C**) Western blot detection of biotinylated proteins in PDI6-TurboID-4Ty parasites. Biotinylated proteins were detected using streptavidin-HRP, TgPDI6 was detected with anti-Ty antibodies, and anti-Actin antibodies were used as a loading control. (**D**) Venn diagram representing the proteins shared in PDI6-TurboID pulldowns. (**E**) Volcano plot illustrating the differential abundance of biotinylated proteins in TgPDI6 samples (repeat 1) compared with RH samples. Biotinylated proteins were filtered based on corrected *P* values of 0.05 and a log2 (fold change) of 3. (**F**) Diagram showing the predicted subcellular localization of biotinylated proteins identified from TgPDI6 samples (repeat 1) using hyperLOPIT data (18). (**G**) Comparison of TgPDI6-enriched proteins with total proteins identified by hyperLOPIT, predominantly located in secretory organelles and the endoplasmic reticulum.

To identify interacting and proximal proteins of TgPDI8 and TgPDI6, we fused the TurboID biotin ligase sequence with a 4Ty tag to the C-terminus of the bait protein at the endogenous locus. The PDI6-TurboID fusion was perfectly trafficked to the ER, whereas the PDI8-TurboID fusion localized to the ER but also displayed partial secretion into the PV (Fig. 4A and B). Despite attempts to introduce the GEEL sequence following TurboID-4Ty or TurboID-3HA, none of these modifications led to exclusive ER localization of the PDI8-TurboID fusion (Fig. 4A). Given the mislocalization of PDI8-TurboID fusion, our study focused primarily on identifying biotinylated protein enrichment associated with TgPDI6. Upon biotin addition, the PDI6-TurboID-4Ty strain showed self-biotinylation of the fusion protein, as detected by streptavidin-FITC but not observed in untreated parasites (Fig. 4B). Western blot analysis further confirmed a significant increase in biotinylated proteins in biotin-supplemented parasites, validating the activity of TurboID *in vitro* (Fig. 4C). Subsequently, liquid chromatography-tandem mass spectrometry (LC-MS/MS) analysis was conducted to identify biotinylated proteins in treated PDI6-TurboID-4Ty parasites. Using a significance cutoff of *P* < 0.05 and a log2 fold change of ≥3, a total of 320 proteins were identified: 310 and 242 proteins in two separate samples, with 232 proteins common to both (Fig. 4D). Hereafter, we designate the list of 320 proteins associated with TgPDI6. Among these, 313 proteins were analyzed for subcellular localization using hyperLOPIT data (Table S2) (18). In addition to ER and apicoplast proteins, the proteins enriched by TgPDI6 primarily include those associated with secretory organelles, such as micronemes, dense granules, and rhoptries (Fig. 4F and G; Fig. S4B). TgPDI8 protein is also significantly enriched, suggesting close spatial proximity to TgPDI6 (Fig. 4E; Fig. S4A). To further evaluate their importance for parasite growth, we analyzed phenotype scores from the Sidik et al. data set (19), categorizing proteins as dispensable (phenotype score > −2), important (−2 to −4), or critical (<−4). Notably, 26% of these proteins were critical, and 19% were important for parasite growth (Table S2). CRMPB was identified as the most significantly enriched protein (Fig. 4E; Fig. S4A), forming a complex with CRMPA, MIC15, and TSP1, critical for parasite invasion (23). The remaining three proteins also exhibited notable enrichment when TgPDI6 was used as bait. Both CRMPA and CRMPB, characterized by multiple cysteine residues, undergo intricate oxidative folding within the ER, likely depending on PDI proteins for proper processing. Furthermore, several microneme proteins (MIC2/3/8/15) and rhoptry neck proteins (RON4/5/8/10) exhibited significant enrichment (Fig. 4E; Fig. S4A), crucial for parasite invasion. Additionally, validated substrates or interacting partners of PDI8 in *Plasmodium falciparum*, such as BiP, PDI11, and PfJ2, were also significantly enriched in our data set (10). In conclusion, the proteins enriched by TgPDI6 are primarily associated with the classical secretory pathway of *T. gondii*.

### Conditional knockdown of TgPDI8 results in the downregulation of secretory protein expression

**Fig 5:**
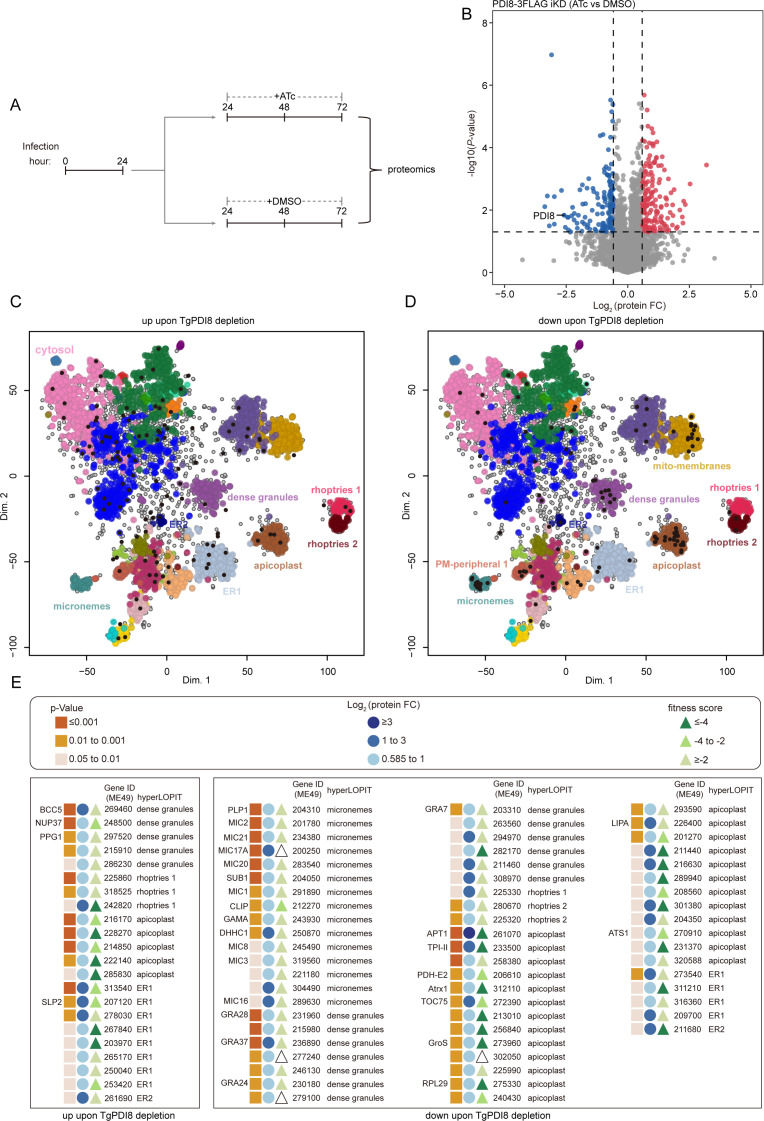
Decreased expression of multiple secretory proteins caused by TgPDI8 depletion. (**A**) Timeline for the collection of proteome samples by treating parasites with ATc and DMSO. (**B**) Volcano plot highlighting the differentially expressed proteins identified from the comparison of ATc versus DMSO in PDI8-3FLAG iKD parasites. The cutoff for differential expression was an adjusted *P* value < 0.05 and ±1.5 fold change. *x*-axis shows log2 fold change, and *y*-axis shows −log10(*P* value). (**C and D**) Diagram showing the predicted subcellular localization of upregulated (**C**) and downregulated (**D**) expression proteins following TgPDI8 depletion using hyperLOPIT data (18). (**E**) Diagram depicting the alteration in expression of secretory proteins and ER proteins upon TgPDI8 depletion. Fitness scores from Sidik et al. (19); HyperLOPIT data from Barylyuk et al. (18). Gene names (left); TGME49 gene IDs (right).

To investigate the impact of TgPDI8 on protein expression, we assessed proteomic changes following treatment with ATc or DMSO for 48 h (Fig. 5A). A comparison of protein expression profiles between ATc- and DMSO- treated parasites revealed 304 significantly differentially expressed proteins, with 167 proteins upregulated and 137 proteins downregulated (Fig. 5B). The data set analysis by Sidik et al. indicated that approximately 46% of these proteins are important for parasite growth (19). Subsequent analysis of differentially expressed proteins using hyperLOPIT data revealed a predominant localization of downregulated proteins in secretory organelles, particularly micronemes and dense granules (Fig. 5D and E) (18). Among these, several MIC proteins, including MIC1/2/3/8/16/17A/20/21, exhibited significant downregulation (Fig. 5E). Given the critical roles of microneme proteins in gliding motility and active invasion, the decreased invasion capability upon TgPDI8 depletion is unsurprising. Furthermore, several apicoplast proteins crucial for parasite growth were downregulated (Fig. 5E). For example, apicoplast phosphate transporter 1, known to traffic through the ER to the apicoplast (24–26), is essential for parasite growth. Our TurboID data also demonstrate that TgPDI6 effectively biotinylates apicoplast proteins. The observed downregulation further suggests that these apicoplast proteins likely depend on ER-localized PDI proteins for processing. In conclusion, depletion of TgPDI8 resulted in reduced expression of proteins localized in micronemes, dense granules, and apicoplast, highlighting its pivotal role in protein processing.

### Transient and unstable nature of interacting proteins with TgPDI8

**Fig 6:**
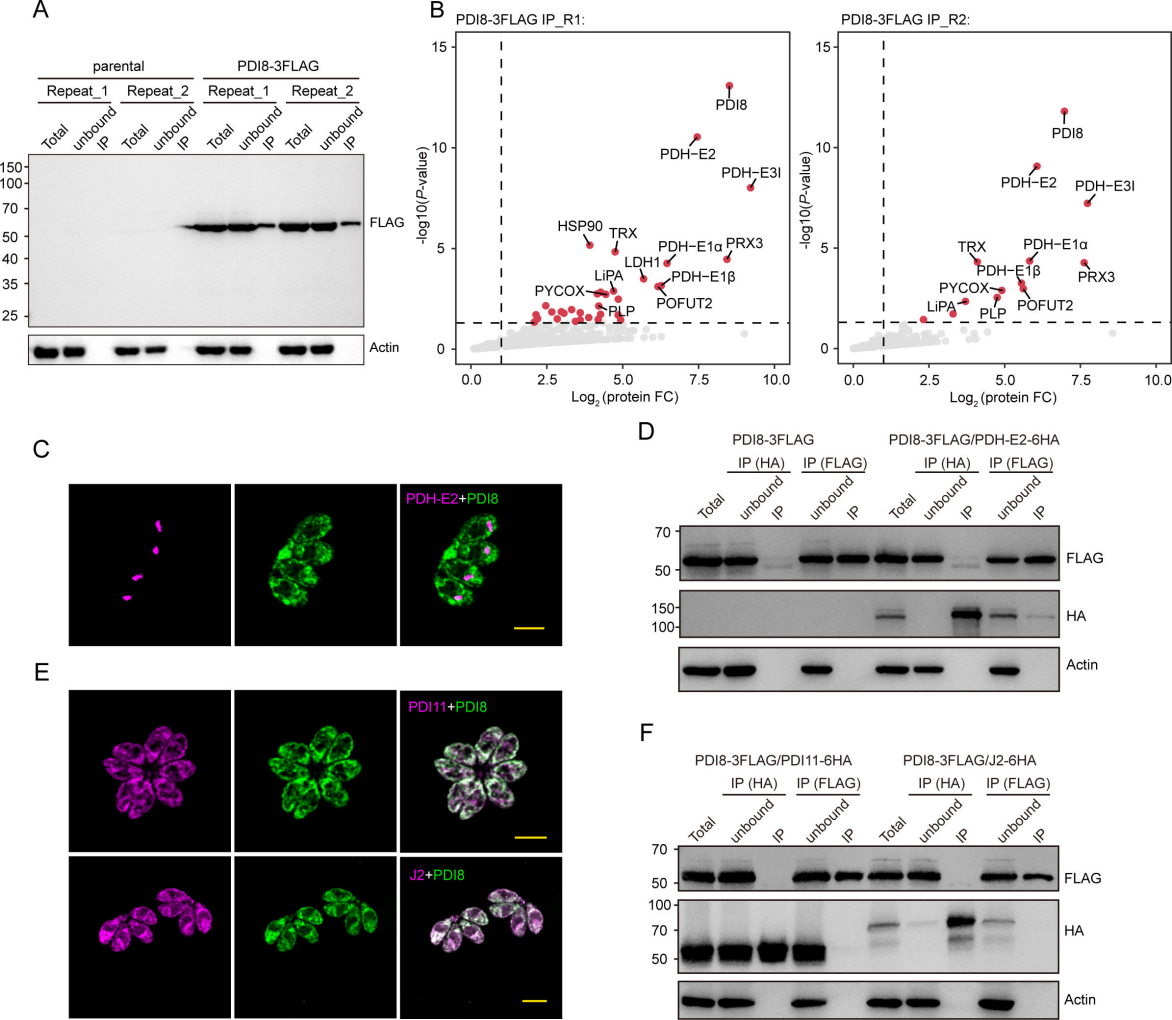
Identification of potential interacting proteins of TgPDI8 using immunoprecipitation. (**A**) Western blot verification of immunoprecipitation (IP) efficiency using anti-FLAG antibodies. Anti-actin antibodies were used as a loading control. (**B**) Volcano plots showing the enriched proteins purified from PDI8-3FLAG parasites using anti-FLAG IP and identified by mass spectrometry. The IP experiment was independently replicated twice. (**C**) IFAs of PDI8-3FLAG/PDH-E2-6HA parasites labeled with anti-HA antibodies (magenta) and anti-FLAG antibodies (green). (**D**) Western blots of protein extracted from PDI8-3FLAG/PDH-E2-6HA parasites, followed by IP using anti-FLAG or anti-HA antibody-coupled beads. TgPDI8 was detected using anti-FLAG antibodies, and TgPDH-E2 was detected using anti-HA antibodies. Anti-actin antibodies were used as a loading control. (**E**) IFAs of PDI8-3FLAG/PDI11-6HA and PDI8-3FLAG/J2-6HA parasites labeled with anti-HA (PDI11 and J2, magenta) and anti-FLAG (PDI8, green) antibodies. (**F**) Western blots of proteins extracted from PDI8-3FLAG/PDI11-6HA and PDI8-3FLAG/J2-6HA parasites, followed by IP using anti-FLAG or anti-HA antibody-coupled beads. TgPDI8 was detected using anti-FLAG antibodies, and TgPDI11 and TgJ2 were detected using anti-HA antibodies. Anti-actin antibodies were used as a loading control.

To identify proteins interacting with TgPDI8, we performed immunoprecipitation experiments using PDI8-3FLLG parasites. TgPDI8-associated proteins were purified using magnetic beads conjugated with FLAG antibodies, followed by LC-MS/MS analysis (Fig. 6A). The enriched proteins primarily included validated apicoplast proteins such as PDH-E1α, PDH-E1β, PDH-E2, PDH-E3I, and LiPA (Fig. 6B), which are known to participate in fatty acid synthesis within the apicoplast (27, 28). Based on hyperLOPIT data (18), two proteins enriched by TgPDI8 were predicted to localize within the ER, including POFUT2 (TGME49_273550) and PLP (TGME49_300350) (Fig. 6B). TgPOFUT2 initiates TSR O-glycosylation and plays an important role in the folding and stabilization of these proteins (29, 30), indicating its potential involvement as a substrate or partner in protein processing alongside PDI proteins. Additionally, two antioxidant enzymes, Trx (TGME49_224060, Golgi) and Prx3 (TGME49_230410, mitochondrion soluble), were significantly enriched by TgPDI8 (Fig. 6B).

To confirm the interaction between TgPDI8 and specific enriched proteins, a 6×HA epitope tag was introduced at the C-terminus of TgPDH-E2 in the PDI8-3FLAG background. Subsequent IFAs revealed that PDH-E2 localizes to the apicoplast (Fig. 6C). Co-immunoprecipitation (Co-IP) assays detected TgPDH-E2 in the products enriched with TgPDI8; however, this was not observed conversely (Fig. 6D). TgPDH-E2 shows significant enrichment when using TgPDI6 as bait, and its downregulation upon TgPDI8 depletion suggests a requirement for processing within the ER. Previous studies suggest that PDI8 may function in a complex with pfJ2 and other ER chaperones to facilitate protein folding (10). To investigate potential interactions in *T. gondii*, we identified two homologous proteins, TgJ2 (TGME49_204480) and TgPDI11 (TGME49_249270), both bearing the canonical ER retention signal H/KDEL, through sequence alignment. Subsequently, a 6HA epitope tag was introduced before the H/KDEL sequence of TgJ2 and TgPDI11 within the PDI8-3FLAG background. IFA and Co-IP were conducted to analyze the subcellular localization and interactions between TgPDI8 and TgJ2 or TgPDI11. Tgf2/TgPDI11-6HA parasites exhibited colocalization with TgPDI8 (Fig. 6E), while no direct interaction was detected between TgPDI8 and TgPDI11 or Tgf2 (Fig. 6F). Additionally, these proteins were not prominently enriched in our IP data, possibly due to the transient and unstable nature of interactions with TgPDI8.

## DISCUSSION

To date, multiple Trx domain-containing proteins have been annotated in the *T. gondii* genome, yet the biological functions and interaction networks of ER-localized members of the Trx superfamily remain largely elusive. Here, we performed a reverse genetic screening to identify ER-localized PDI proteins in *T. gondii*. Our findings demonstrate that conditional knockdown of TgPDI8 severely impairs parasite growth *in vitro*. Complementation with mutant TgPDI8 confirmed the functional importance of the CXXC active site cysteines. Our investigations using TurboID-based proximity labeling provided evidence of TgPDI8’s close association with secretory proteins. Furthermore, quantitative proteomics analysis revealed significant alterations in the expression of secretory proteins following TgPDI8 depletion.

ER-localized proteins typically feature classical ER retention signals (H/KDEL) (31). In this study, we identified two proteins: TgPDI8, which carries the GEEL sequence, and TgPDI6, containing the RDEL sequence. The C-terminal epitope tagging of TgPDI6 with 6Ty did not alter its ER localization, whereas TgPDI8 was observed in both the ER and PV. Subsequent attempts to introduce various tags before the GEEL sequence of TgPDI8 successfully established an endogenously tagged strain, PDI8-3FLAG, exclusively localized to the ER. Using a CRISPR/Cas9-mediated strategy, we replaced the TgPDI8 promoter to generate an ATc-inducible TgPDI8 regulatable strain (PDI8-3FLAG iKD), specifically localized to the ER. However, multiple TgPDI8 transgenic strains, including a DiCre-induced conditional knockdown strain, a 6Ty-tagged complementation strain, and PDI8-TurboID fusion strains, exhibited dual localization in both the ER and PV. This dual localization pattern may be attributed to the introduction of a larger tag at the C-terminus of PDI8. Furthermore, it is worth noting that TgPDI8 carries an imperfect ER-retention signal (GEEL), potentially contributing to its secretion. Although mislocalization of 6Ty-fused TgPDI8 does not fully impair its function, complementation experiments confirm that its altered localization adversely impacts parasite growth.

Conditional knockdown of TgPDI8 resulted in growth defects, particularly affecting proliferation and invasion capabilities in *T. gondii*. This finding is consistent with previous studies indicating the crucial role of PDI8 in mediating parasite invasion, as evidenced by the use of PDI8 inhibitors (15, 16). TgPDI8 harbors two conserved Trx domains, each featuring classical CXXC active site cysteines. *In vitro* studies with recombinant protein have confirmed the essential role of these CXXC active site cysteines in PDI8 enzymatic activity (9, 15). Previous attempts to validate substrate binding through overexpression of mutant PDI8 protein in malaria parasites, with cysteine-to-alanine mutations in the Trx domain active sites, were unsuccessful (10). To our surprise, we successfully generated wild-type and mutant TgPDI8-complemented strains, further confirming the crucial role of CXXC in parasite growth. Mutation of either CXXC active site cysteine significantly affects parasite growth but is non-lethal. However, mutation of both CXXC active site cysteines completely blocks plaque formation, thereby confirming their synergistic role.

Secretory proteins undergo oxidative folding in the ER to achieve their native state. ER-localized proteins fused with TurboID can biotinylate secretory proteins, as confirmed by previous studies. For example, Kim et al. utilized the *in situ* secretory protein labeling via ER-anchored TurboID method to label canonical secretory proteins both *in vivo* and *in vitro* (32). Moreover, the utilization of an ER retention signal (KDEL) enables functional investigations of secretory and apicoplast proteins in *Plasmodium* by appending the KDEL sequence to their C-terminus, thereby preventing their secretion (33). Our TurboID data provided candidate proteins suitable for this approach. Additionally, our findings suggest significant enrichment of several rhoptry neck proteins (RON2, RON4, RON5, RON8, and RON10) when using TgPDI6 as bait. Notably, these rhoptry neck proteins, particularly RON2/4/5/8, interact with the microneme protein AMA1 and play essential roles in the formation of the moving junction, crucial for parasite invasion (34–37). Quantitative proteomics analysis also demonstrated a reduction in the expression of secretory proteins, particularly MIC proteins crucial for host cell attachment, following TgPDI8 depletion (38–41).

Previous studies have indicated that PfJ2 interacts with other ER-resident chaperones like PfPDI8 and PfBiP, to facilitate protein folding (10). Although these proteins were exclusively enriched in our TurboID data set, they were not detected in the IP data set. Subsequent Co-IP experiments confirmed the absence of interaction between these proteins and TgPDI8. One plausible explanation is the potential instability of their binding interactions with TgPDI8, making their capture challenging through IP methods. DVSF has shown efficacy in trapping and identifying Trx-domain protein substrates (42–44), but its high toxicity necessitates the exploration of safer alternatives.

In summary, our study demonstrates the crucial role of ER-localized TgPDI8 in supporting *T. gondii* growth. Future research will aim to identify additional ER-localized proteins and elucidate their roles in parasite growth. Given the conservation of this gene across apicomplexan parasites, it provides promising prospects for the future development of novel antiparasitic drugs.

## MATERIALS AND METHODS

### Parasites and cell culture

The RH Δ*Ku80*Δ*hxgprt*, DiCre RH, and RH TIR1-3Flag lines were propagated in HFF cells obtained from ATCC. Infected cells were maintained in Dulbecco’s modified Eagle’s medium (Macgene) supplemented with 2% fetal bovine serum (Sigma-Aldrich) at 37℃ in a 5% CO2 atmosphere. Parasites were passaged every 3–4 days on HFF cells following standard procedures.

### Bioinformatic analyses

The protein sequences used in this study were obtained from ToxoDB (https://toxodb.org/toxo/app) (45). Functional domains were predicted using SMART (http://smart.embl.de/). Homologous protein sequences to TgPDI8 were obtained from VEuPathDB (https://veupathdb.org/veupathdb/app) (46). The alignment of protein sequences from TgPDI8 and its homologs was performed using ClustalW. A phylogenetic tree was generated via neighbor-joining method, and the Newick format tree was visualized using MEGA11 (47). Candidate proteins identified through TurboID-based proximity labeling were mapped on the high-resolution spatial proteome map of *T. gondii* using the hyperLOPIT data set (https://proteome.shinyapps.io/toxolopittzex/) (18).

### Generation of transgenic *T. gondii* strains

Primers and plasmids used or constructed in this study are listed in Table S1.

### CRISPR/Cas9-mediated gene tagging

The PDI8-3FLAG parasites were generated by introducing a 3xFLAG tag at the C-terminus of the TgPDI8 using the modified plinker-3FLAG-CAT plasmid. The ER retention sequence GEEL of TgPDI8 was inserted between the FLAG tag and stop codon of the plinker-3FLAG-CAT plasmid, generating the plinker-3FLAG(GEEL)-CAT plasmid. For C-terminal epitope tagging of TgPDI8, specific 59-bp PCR primers were designed containing 42-bp fragments upstream of the TgPDI8 GEEL sequence and downstream of the Cas9 break site to amplify PCR products from the plinker-3FLAG (GEEL)-CAT plasmid. RH Δ*Ku80*Δ*hxgprt* parasites were co-transfected with the Cas9-expressing pU6-PDI8 (3′UTR) plasmid and PCR-purified products, followed by selection with chloramphenicol (34 mg/mL). Using the plinker-6HA-DHFR plasmid as a template, the same strategy was employed to construct the plinker-6HA-HDEL-DHFR and plinker-6HA-KDEL-DHFR plasmids. To generate PDI8-3FLAG/PDH-E2-6HA, PDI8-3FLAG/PDI11-6HA, and PDI8-3FLAG/f2-6HA lines, PDI8-3FLAG parasites were co-transfected with specific CRISPR/Cas9 plasmids and purified PCR products containing 6HA-H/KDEL-DHFR or 6HA-DHFR cassette, followed by selection with 3 µM pyrimethamine. Stable transfectants were cloned by limiting dilution and confirmed by IFA and western blot analysis.

### CRISPR-Cas9-mediated gene knockdown (AID/TIR1, TATi-TetR, and 4U1/DiCre)

The PDI8-AID-3HA line was generated using the AID system. Specific 59-bp PCR primers, containing 42-bp fragments upstream of the TgPDI8 translation stop codon sequence and downstream of the Cas9 break site, were designed to amplify PCR products from the plinker-AID-3HA-DHFR plasmid. TIR1-3FLAG RH parasites were co-transfected with Cas9-expressing pU6-PDI8 (3′UTR) plasmid and purified PCR products, followed by selection with pyrimethamine. The PDI8-3FLAG iKD line was generated using the Tet-inducible system. Specific 59-bp primers were designed for PCR amplification from the p-T8-TATi-1-HX-TetO7SAG1 plasmid, containing 42-bp fragments upstream of the Cas9 break site and downstream of the start codon of TgPDI8. PDI8-3FLAG parasites were co-transfected with the Cas9-expressing pU6-PDI8 (5′UTR) plasmid and PCR-purified products, followed by selection with 25 µg/mL mycophenolic acid and 50 µg/mL xanthine. To generate PDI6-6Ty-4U1, PDI8-6Ty-4U1, and PDI8-6Ty-GEEL-4U1 lines, DiCre RH parasites were co-transfected with specific CRISPR/Cas9 plasmids and purified PCR products containing 6Ty-4U1-HXGPRT or 6Ty-GEEL-4U1-HXGPRT cassette based on HXGPRT selection. Stable transfectants were cloned through limiting dilution and confirmed using IFA and western blot analysis.

### Generation of ΔPDI6 line

The CRISPR/Cas9 plasmid was co-transfected with a gene-specific knockout plasmid containing a floxed DHFR-TS* selectable marker flanked by gene-specific 5′ and 3′ homology arms. Stable transfectants were obtained through limiting dilution and validated by diagnostic PCR.

### Generation of TgPDI8-complemented lines

The pUPRT::Tub-GOI-6Ty was used as a template to replace the promoter and insert the GEEL sequence between the 6Ty tag and stop codon, resulting in the pUPRT:: PDI8-GOI-6Ty-GEEL plasmid. Wild-type TgPDI8 cDNA was then cloned into this modified plasmid. The Cas9-expressing pU6-UPRT plasmid and PCR-purified products were co-transfected into PDI8-3FLAG iKD parasites. To investigate the effects resulting from mislocalization of 6Ty-fused TgPDI8, the 6Ty tag was removed using pUPRT:: PDI8-GOI-6Ty-GEEL plasmid as a template. Mutant TgPDI8 cDNA was then inserted into this plasmid. Transgenic parasites were selected with 5-fluorodeoxyuridine (5 µM) and cloned by serial limiting dilution. Confirmation of complemented lines was carried out through IFA, western blot analysis, or diagnostic PCR.

### Generation of PDI8 and PDI6-TurboID fusions

The plinker-TurboID-3HA-DHFR and plinker-TurboID-4Ty-DHFR plasmids were used as templates for inserting the GEEL sequence between the 3HA or 4Ty tag and stop codon, resulting in the plinker-TurboID-3HA-GEEL-DHFR and plinker-TurboID-4Ty-GEEL-DHFR plasmids. To generate PDI8- and PDI6-TurboID fusions, specific 59-bp PCR primers were designed containing 42-bp fragments upstream of the TgPDI8 or TgPDI6 translation stop codons and downstream of the Cas9 break site to amplify PCR products from the plasmids containing the TurboID-3HA-GEEL-DHFR or TurboID-4Ty-GEEL-DHFR cassette, based on DHFR selection. Stable transfectants were isolated by limiting dilution and confirmed by IFA and western blot analysis.

### Antibodies

Primary antibodies used in this study included rabbit anti-GAP45 (48), rabbit anti-BiP, mouse anti-MIC2 (49), mouse anti-GRA3, mouse anti-ACP, rabbit anti-RON2, mouse mAb DG52 anti-SAG1, mouse anti-HA (Sigma-Aldrich), mouse anti-FLAG (Sigma-Aldrich), and mouse anti-Ty. IFAs employed secondary antibodies labeled with Cy3/FITC-conjugated goat anti-mouse IgG or Cy3/FITC-conjugated goat anti-rabbit IgG. For western blot analysis, primary antibodies included mouse anti-HA, mouse anti-FLAG, mouse anti-Ty, and mouse anti-TgActin (50), and secondary antibodies consisted of HRP-conjugated goat anti-mouse or rabbit IgG (Proteintech, Chicago, IL, USA).

### Immunofluorescence assay

Freshly harvested parasites were inoculated onto HFF monolayers grown on coverslips. The infected cells were fixed with 4% paraformaldehyde for 30 min, permeabilized with 0.25% Triton X-100 (Sigma-Aldrich) for 30 min, and blocked with 3% bovine serum albumin for 30 min. Following this, coverslips were incubated with primary antibodies for 1 h, followed by three washes with PBS. Secondary antibodies and Hoechst 33258 were then incubated for 1 h and washed three times with PBS. The infected monolayers were observed by a Leica confocal microscope (TCS SP52, Leica, Germany) at a magnification of 63×. High-content imaging and analysis were performed with the LAS AF lite 2.2.0 software.

### Immunoblotting

Protein samples were lysed in RIPA buffer (50 mM Tris [pH 7.4], 150 mM NaCl, 1% Triton X-100, 1% sodium deoxycholate, and 0.1% SDS; Beyotime) supplemented with the protease inhibitor PMSF, resolved by SDS-PAGE, and transferred onto nitrocellulose membranes. Primary and secondary antibodies were incubated for 1 h each at room temperature. Protein detection was achieved using chemiluminescence for visualization.

### Plaque assay

Freshly harvested parasites were inoculated onto HFF monolayers (70 parasites per well in 12-well plates). For Tet-inducible knockdown lines, infected cells were treated with ATc or vehicle (DMSO, 1:1,000). For DiCre-mediated U1 knockdown lines, infected cells were treated with 50 nM rapamycin (MCE) or DMSO for 4 h before washout. After 7 days post-infection, infected cells were fixed in 4% PFA for 20 min at room temperature. Staining was performed for 1 h at room temperature, followed by PBS washing and overnight drying. Plaque formation was assessed by counting the areas of clearance by parasites.

### Intracellular replication assay

For the PDI8-3FLAG iKD line, infected cells were pre-incubated with ATc or DMSO for 24 h. Each well of a 12-well plate was then inoculated with 10^5^ freshly isolated tachyzoites, followed by washing to remove non-invaded tachyzoites at 1 h post-infection. After 24 h post-infection, IFAs were performed using anti-GAP45 antibodies to stain the parasites. The number of tachyzoites per vacuole was counted, with at least 100 vacuoles analyzed per sample in three independent experiments.

### Invasion assay

Parasite invasion assays utilized a differential staining method to distinguish between intracellular and extracellular parasites. Following 48 h of growth in ATc or DMSO, intracellular parasites were collected and inoculated onto HFF monolayers (10^6^ parasites/well) grown on coverslips in 24-well plates for 20 min. After thorough washing, coverslips were first stained with mouse anti-SAG1 to label extracellular parasites. Subsequently, permeabilization with 0.25% Triton X-100 allowed staining with rabbit anti-GAP45 antibodies to label both intracellular and extracellular parasites. Following PBS washes, secondary antibodies conjugated with FITC (goat anti-mouse IgG) and Cy3 (goat anti-rabbit IgG) were applied. Thirty fields were scored for each sample, and all experiments were independently replicated three times.

### Quantitative label-free mass spectrometry

Freshly isolated parasites were inoculated onto HFF monolayers in T-75 flasks and then washed to remove non-invaded parasites after 1 h post-infection. Infected cells were subsequently treated with ATc or DMSO for 48 h. Each protein sample was extracted from an equivalent number (2 × 10^8^) of *Toxoplasma* tachyzoites, quantified by Bradford method, and assessed through SDS-PAGE. Subsequently, 50 μg of parasite proteins was supplemented with DTT to a final concentration of 10 mM and subjected to a 1-h incubation at 37°C, followed by treatment with iodoacetamide for 30 min at room temperature. Following this, all samples were diluted fourfold with 25 mM ammonium bicarbonate buffer and enzymatically digested overnight at 37°C with trypsin (trypsin: protein = 1:50). The digested peptides were retrieved, desalted, and concentrated for subsequent detection. Eluted peptides were analyzed using a Q Exactive HF-X mass spectrometer (Thermo Fisher Scientific, USA). Raw data files were processed with MaxQuant software and matched against the ToxoDB *Toxoplasma* Genomics Resource (ToxoDB-68_TgondiiME49_AnnotatedProteins.fasta). Differentially expressed proteins with a fold change > 1.5 and corrected *P* values < 0.05 were further analyzed.

### Biotinylation approaches

For biotin-labeling experiments, untagged RH parasites or parasites expressing TurboID-tagged PDI6 were used to infect HFF monolayers. At 36 h post-infection, biotin (Sigma-Aldrich) was added to a final concentration of 160 µM and cultured for an additional 4 h until natural egress occurred. The harvested parasites were then washed three times with cold PBS to remove excess biotin. Biotinylated proteins were purified using streptavidin magnetic beads (Thermo Fisher Scientific, TG268480); 10% of the beads was reserved for western blot analysis. The remaining beads were loaded onto an SDS-PAGE gel, and bands were excised for mass spectrometry analysis. A detailed protocol for purifying biotinylated proteins was performed following established procedures (51).

### Immunoprecipitation and co-immunoprecipitation

Freshly lysed parasites from four T-75 flasks per sample were collected and lysed in cold IP buffer (50 mM Tris [pH 7.4], 150 mM NaCl, and 1% NP-40, protease inhibitor cocktail). Following four cycles of freeze/thaw, the lysate was sonicated on ice with a total pulse time of 3 min (2 seconds on and 3 seconds off). The sonicated samples were then centrifugated at 11,000 × *g* for 15 min at 4 °C and incubated overnight at 4°C with mouse anti-FLAG antibodies. Following the overnight incubation, protein A/G magnetic beads (MCE) were added and incubated for an additional 3 h. Subsequently, the beads were washed four times with cold IP buffer and two times with cold PBS. A 10% fraction of the beads was resuspended in SDS-PAGE sample buffer for western blot analysis. The remaining fraction of beads was loaded onto an SDS-PAGE gel, and bands were excised for mass spectrometry analysis. For the co-immunoprecipitation experiments, freshly lysed parasites were purified and lysed in cold IP buffer. The experimental procedure followed was consistent with the IP method described above. The sonicated samples were individually incubated with anti-FLAG antibodies and anti-HA antibodies and analyzed by western blotting.

### LC-MS/MS acquisition and data analysis

Gel samples from all IP and TurboID experiments were digested with trypsin, followed by peptide desalting using C18 Cartridges (Empore SPE Cartridges C18 [standard density], bed I.D. 7 mm, volume 3 mL, Sigma), and reconstitution in 40 µL of 0.1% (vol/vol) formic acid. LC-MS/MS analysis was performed on the Nanoelute (Bruker) coupled to timsTOF Pro mass spectrometry (Bruker). Precursors and fragments were analyzed using the TOF detector, with full mass scans acquired over an *m*/*z* range of 100–1,700. The MS raw files were processed with MaxQuant (version 1.6.14) for peptide identification and quantitative analysis. Searches were mapped against the *T. gondii* database (ToxoDB-56_TgondiiGT1_AnnotatedProteins.fasta). Quantitative analysis followed established protocols (52), with appropriate settings for *P* values and log2 (fold change) significance cutoffs determined for both IP and TurboID experiments.

### Quantification and statistical analysis

All quantification data are presented as mean ± SD. Statistical significance was calculated by unpaired two-tailed Student’s *t*-test, and all analyses were performed with GraphPad Prism 8.0 software (https://www.graphpad.com/). Differences were considered statistically significant when *P* < 0.05. Specific details regarding the number of biologically independent replicates (*n*) and statistical methods can be found in the figure legends of both the main text and supplemental materials.

## Data Availability

The data sets generated for this study are included within the article and supplemental files. The mass spectrometry proteomics data have been deposited to the ProteomeXchange Consortium (http://proteomecentral.proteomexchange.org) with the accession number PXD053468.

## SUPPLEMENTAL MATERIAL

Supplemental Figures mbio.02051-24-s0001.docx Figures S1 to S4.

Table S1 mbio.02051-24-s0002.xlsx Oligonucleotides, plasmids, and strains used for this study.

Table S2 mbio.02051-24-s0003.xlsx Analysis of neighboring proteins of TgPDI6 using a proximity-dependent biotinylation approach.

Table S3 mbio.02051-24-s0004.xlsx Identification of differentially expressed proteins following TgPDI8 depletion.

Table S4 mbio.02051-24-s0005.xlsx Analysis of interacting proteins associated with TgPDI8.

ASM does not own the copyrights to Supplemental Material that may be linked to, or accessed through, an article. The authors have granted ASM a non-exclusive, world-wide license to publish the Supplemental Material files. Please contact the corresponding author directly for reuse.
