# Supplementary material for: Protein disulfide isomerase PDI8 is indispensable for parasite growth and associated with secretory protein processing in Toxoplasma gondii
Source: mBio. 2024 Aug 20;15(9):e02051-24. doi: 10.1128/mbio.02051-24 (PMC11389393; doi:10.1128/mbio.02051-24)
Supplement: Supplemental Figures — Figures S1 to S4. [file mbio.02051-24-s0001.docx]

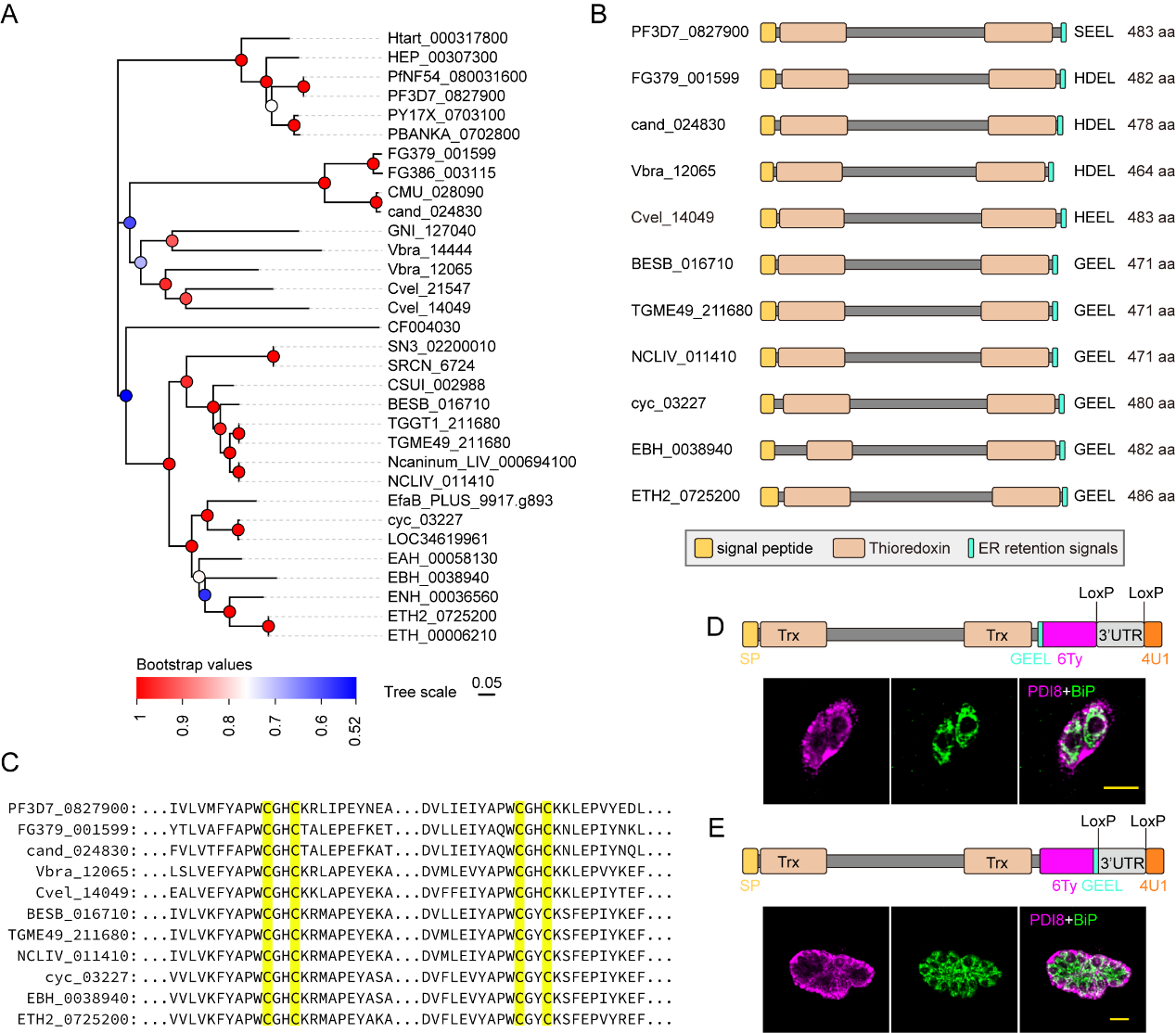


**Fig S1** **Phylogeny and domain architecture of TgPDI8**

(A) Phylogenetic analysis of the entire coding sequence of TgPDI8. Alignment was conducted using ClustalW, and bootstrap values were calculated from 10,000 trials. (B) Predicted domain structure of TgPDI8 and its homologous proteins in other species, showing signal peptide (SP), two thioredoxin domains (Trx), and C-terminal ER retention signals (GEEL). Domain predictions were performed using SMART. (C) Alignment of two conserved cysteine residues in the selected species as depicted in Fig 1B, with residues critical for redox activity highlighted in yellow. (D-E) Immunofluorescence assays (IFAs) of PDI8-6Ty-4U1 (D) and PDI8-6Ty-GEEL-4U1 (E) parasites using anti-Ty antibodies (magenta, D and E). Anti-BiP antibodies served as an ER marker (green, D and E). Scale bars = 5 µm.


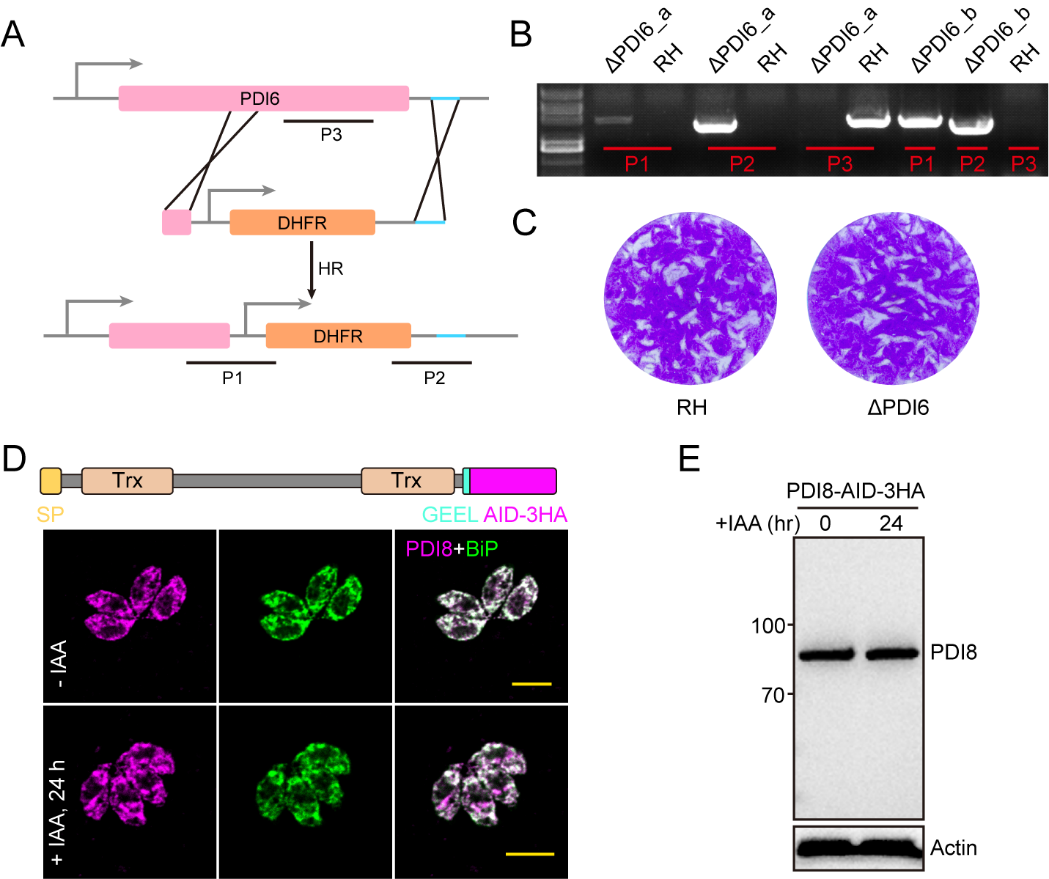


**Fig S2** **TgPDI6 is not essential for parasite growth**

(A) Strategy for the generation of ΔPDI6 parasites. (B) Diagnostic PCR demonstrating homologous integration and gene knockout in two representative clones (ΔPDI6_a and b) compared to the RH parasites. (C) Plaque formation by RH and ΔPDI6 parasites on HFF monolayers after 7 days. (D-E) IFAs and western blot analysis of PDI8-AID-3HA parasites after treatment with 500 µM IAA or vehicle (EtOH 1:1000). Parasites were stained with Ty antibodies (magenta) and the ER marker BiP (green). Scale bars=5 µm. Western blot depicts the signal of TgPDI8 with or without IAA treatment, with TgActin antibodies used as a loading control.


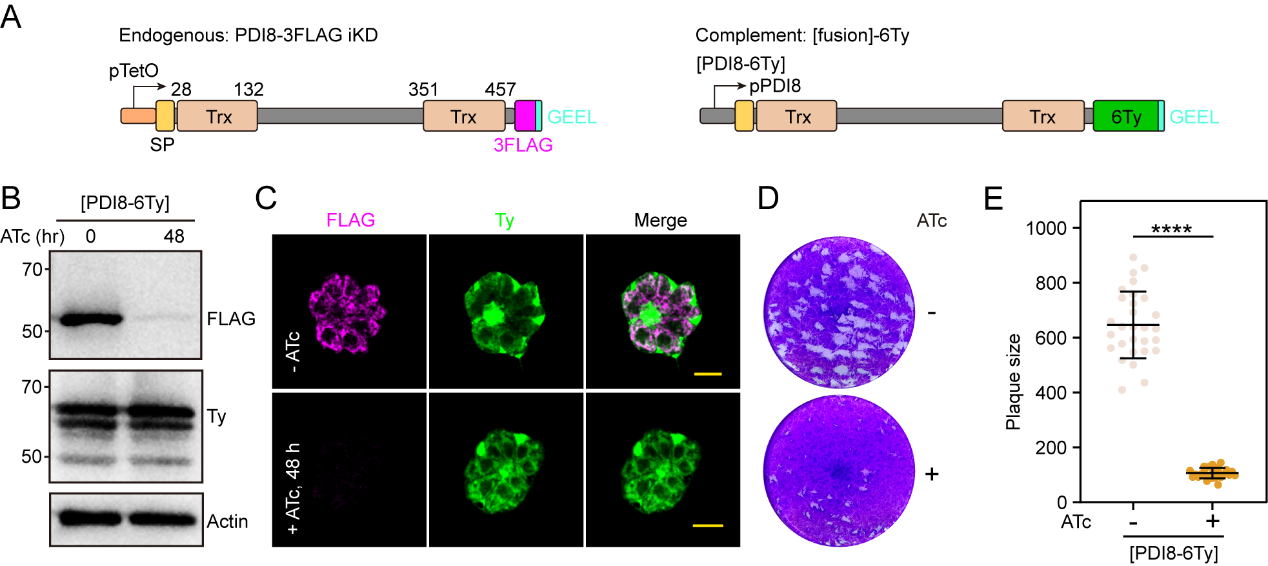


**Fig S3 Impact of mislocalized 6Ty-fused TgPDI8 on parasite growth**

(A) Schematic representation of inserting a second copy of wild-type TgPDI8 into the UPRT locus in the regulatable PDI8-3FLAG iKD background, driven by the native TgPDI8 promoter. (B) Western blot analysis showing the expression of the second copy of wild-type TgPDI8 using anti-Ty antibodies in the absence or presence of ATc. Endogenous TgPDI8 was detected using anti-FLAG antibodies, with antibodies against TgActin used as a loading control. (C) IFAs of intracellular [PDI8-6Ty] parasites treated with ATc or DMSO for 48 h. Parasites were detected with anti-FLAG (magenta) and anti-Ty (green) antibodies. Scale bars = 5 µm. (D) Plaque formation by [PDI8-6Ty] parasites growing on HFF monolayers for 7 days treated with ATc or DMSO. (E) Quantification of plaques corresponding to D, mean ± SD (n = 3). Statistical evaluation was conducted using an unpaired t- test (ATc versus DMSO), p values: **** < 0.0001.


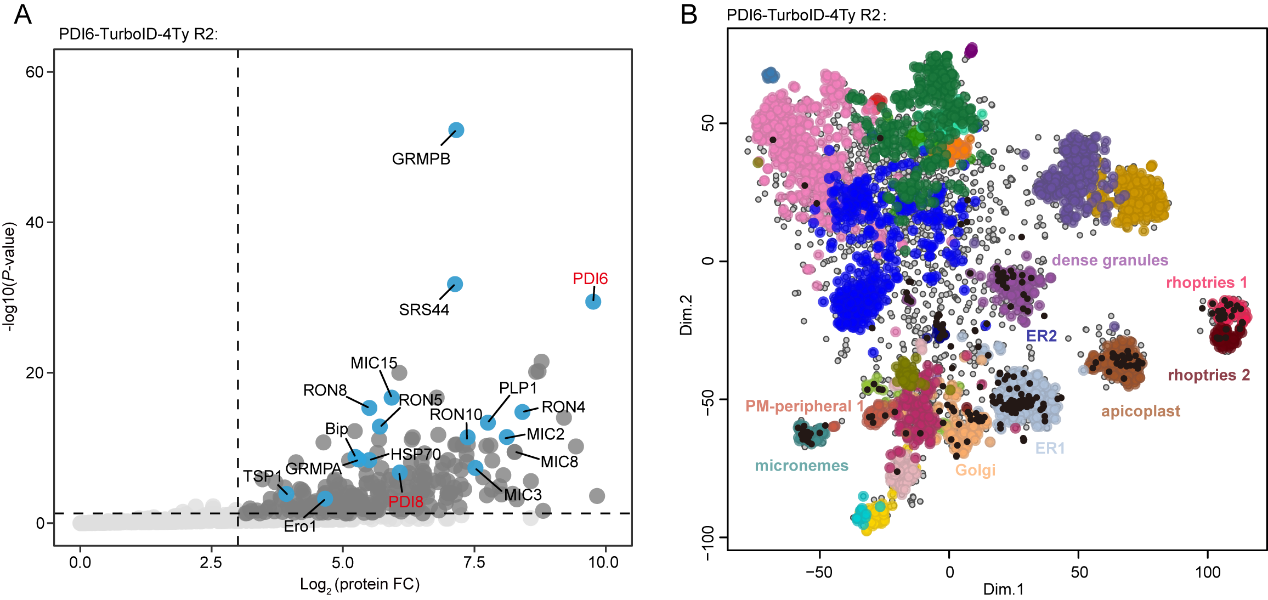


**Fig S4** **Subcellular localization of biotinylated proteins enriched by TgPDI6**

(A) Volcano plot illustrating the differential abundance of biotinylated proteins in TgPDI6 samples (repeat 2) compared to RH samples. Biotinylated proteins were filtered based on corrected *P*-values of 0.05 and a log2 (fold change) of 3. (B) Diagram showing the predicted subcellular localization of biotinylated proteins identified from TgPDI6 samples (repeat 2) using hyperLOPIT data.
